# Investigation of alterations in retrobulbar hemodynamics and their correlation with choroidal thickness after strabismus surgery

**DOI:** 10.1186/s12886-024-03363-3

**Published:** 2024-02-27

**Authors:** Aslihan Uzun, Hilal Altas, Asena Keles Sahin

**Affiliations:** 1 Department of Ophthalmology, Training and Research Hospital, Ordu University, Ordu, 52000 Turkey; 2 Department of Radiology, Training and Research Hospital, Ordu University, Ordu, Turkey

**Keywords:** Choroidal thickness, Color Doppler Ultrasonography, Retrobulbar blood flow, Strabismus surgery

## Abstract

**Background:**

To evaluate the changes in retrobulbar circulation after strabismus surgery and to assess the relationship of these changes with choroidal thickness (CT).

**Methods:**

This prospective study included 26 eyes of 26 patients who underwent strabismus surgery and 15 eyes of 15 healthy individuals as control group. The patients who had single horizontal muscle surgery were included in Group 1 (*n* = 14); and those who had surgery on both horizontal muscles were included in Group 2 (*n* = 12). Peak systolic velocity (PSV), end-diastolic velocity (EDV), resistive index (RI) and pulsatility index (PI) of ophthalmic artery (OA), posterior ciliary artery (PCA), and central retinal artery (CRA) were measured using Color Doppler Ultrasonography. Subfoveal CT was measured via Optical Coherence Tomography. All measurements were obtained preoperatively, at 1st week and 1st month after surgery.

**Results:**

There were no differences regarding preoperative blood flow velocity parameters among the groups. OA RI increased significantly at 1st week and 1st month after surgery in Group 1 and 2 (*P* = 0.029 and *P* = 0.045, respectively). There was a significant increase in PCA PSV at 1st week in Group 1 (*P* = 0.002). There was no difference between the mean preoperative and postoperative CT among the 3 groups. A negative correlation between the percentage changes of CT and CRA EDV was found in Group 2 (*P* = 0.011).

**Conclusion:**

Single and double horizontal rectus muscle surgery have a measurable effect on retrobulbar hemodynamics but these changes do not correlate with CT.

## Background

Anterior segment ischemia (ASI) is a rare but important complication of extraocular muscle surgery with an incidence ranging from 1/13,000 to 1/30,000 [1]. Advanced age, hyperviscosity, systemic vascular diseases, hematologic disorders, carotid-cavernous fistula, and 360^O^ scleral buckling surgery are the risk factors for ASI [2]. Three or more rectus muscle surgery in a single operation, vertical rectus muscle surgery, or a second strabismus surgery within the six months of a previous extraocular muscle surgery may also increase the risk of ASI [1]. Anterior ciliary arteries are not preserved during a conventional strabismus surgery and reestablishment of flow in these arteries is not possible after surgery. Increased blood flow in the long posterior ciliary arteries compensates the interrupted anterior segment circulation [3]. Therefore, some alterations in retrobulbar hemodynamics may occur to supply the increased demand of the anterior segment after strabismus surgery. Monitoring the retrobulbar circulation may ease to understand the exact underlying pathophysiology of ASI.

In 1989, Erickson et al. studied normal and abnormal orbits via Color Doppler Imaging (CDI) and reported that CDI may be helpful in the evaluation of orbital vascular diseases [4]. Since then, CDI has been increasingly used to evaluate the changes in retrobulbar circulation and ocular perfusion [5]. Although the blood flow parameters of the anterior and long posterior ciliary arteries cannot be evaluated directly via CDI, alterations in retrobulbar circulation following strabismus surgery may reflect the changes in anterior segment circulation [6]. Since posterior ciliary artery also provides blood supply to the choroid in addition to the anterior segment, a change in the choroidal thickness (CT) may also be expected after strabismus surgery. CDI of the alterations in retrobulbar blood flow and understanding their relationship with CT may help ophthalmologists predict the ASI risk and take measures to prevent ASI in patients undergoing conventional strabismus surgery.

The aim of this study was to investigate the alterations in retrobulbar circulation after strabismus surgery using Color Doppler Ultrasonography (CDU) and to assess the correlation of these changes with CT. Although there have been previous studies assessing the effect of strabismus surgery on retrobulbar hemodynamics, this is the first study investigating not only the alterations in retrobulbar circulation but also the relationship between CT and these changes.

## Methods

Twenty-six eyes of 26 patients who underwent horizontal strabismus surgery at Ordu University, Training and Research Hospital, Ophthalmology Department between March 2022 and September 2022 were included in this prospective study. Fifteen eyes of 15 age- and sex-matched healthy individuals were assigned as control group. This study was conducted in accordance with the tenets of the Declaration of Helsinki and was approved by Ordu University, Clinical Research Ethics Committee (Approval Number: 2021/220). Written informed consents were obtained from parents or legal guardians of all participants.

This study included patients between 5 and 15 years of age, without a history of previous extraocular muscle surgery. Patients with systemic diseases, cardiovascular abnormalities, craniofacial anomalies, ophthalmologic disorders including nystagmus, retinal anomalies, glaucoma, nystagmus, orbital pathology, sensory or restrictive strabismus were excluded. Patients with a history of prematurity, ocular trauma, previous ocular surgery, and who were unable to cooperate with Optical Coherence Tomography (OCT) or CDI were also excluded.

A detailed ophthalmologic evaluation including best-corrected visual acuity, orthoptic evaluation, slit-lamp biomicroscopy, dilated fundus examination and cycloplegic refraction was performed in all participants. The near and distance deviation angles were measured with alternate prism cover test and recorded as prism diopters. Any abnormal head position or extraocular muscle under/overaction, and nine cardinal positions of gaze were also evaluated.

The patients who had strabismus surgery were divided into 2 groups. Group 1 included 14 eyes of 14 patients (9 patients with esotropia and 5 patients with exotropia) who underwent single horizontal rectus muscle surgery. In Group 1, medial rectus (MR) recession was performed in 9 patients and lateral rectus (LR) recession was performed in 5 patients. Group 2 consisted of 12 eyes of 12 patients (8 patients with esotropia and 4 patients with exotropia) who were operated on both horizontal rectus muscles. In Group 2, MR recession and LR resection were performed in 8 eyes, whereas LR recession and MR resection were performed in 4 eyes. None of the patients underwent vertical rectus or oblique muscle surgery.

### Surgical procedure

All patients underwent strabismus surgery under general anaesthesia by the same experienced surgeon (AU). In Group 1, the recession of bilateral MR or LR muscles were performed in patients with esotropia and exotropia, respectively. In Group 2, MR recession and LR resection were performed in esotropic patients whereas LR recession and MR resection were performed in exotropic patients. The amount of surgery was planned according to the preoperative deviation angle, based on the surgical tables provided by American Academy of Ophthalmology [7]. Following a fornix-based conjunctival incision, the horizontal rectus muscle was identified and hooked. The muscle was secured with an absorbable double-armed 6–0 vicryl suture as close as possible to the insertion for recession surgery, or at a predetermined distance from the insertion for resection surgery, and then disinserted from its insertion. The detached muscle was then reattached to the sclera in the recession surgery or anchored to its original insertion in the resection procedure. An absorbable 8–0 vicryl suture was used for conjunctival closure. Topical moxifloxacin (0.5%) and dexamethasone (0.1%) fixed combination (Moxidexa®, Abdi Ibrahim Pharmaceuticals) was prescribed five times daily for 2 weeks, postoperatively.

### Choroidal imaging

Choroidal images were taken before pupillary dilation via spectral-domain OCT (SD-OCT; Cirrus HD-OCT 4000, Carl Zeiss Meditec Inc., Dublin, CA, USA) with enhanced depth imaging (EDI) mode by the same experienced technician. The scans only with a signal strength of ≥ 7/10 were recorded. Subfoveal CT was obtained by the measurement of perpendicular distance from the outer portion of the hyperreflective line corresponding to the retinal pigment epithelium to the inner surface of the sclera. All CT measurements were performed independently by two masked ophthalmologists (AKS and AU) using the Cirrus HD-OCT software caliper, and the average measurements were taken for the analysis.

### Color doppler imaging (CDI)

**Fig. 1:**
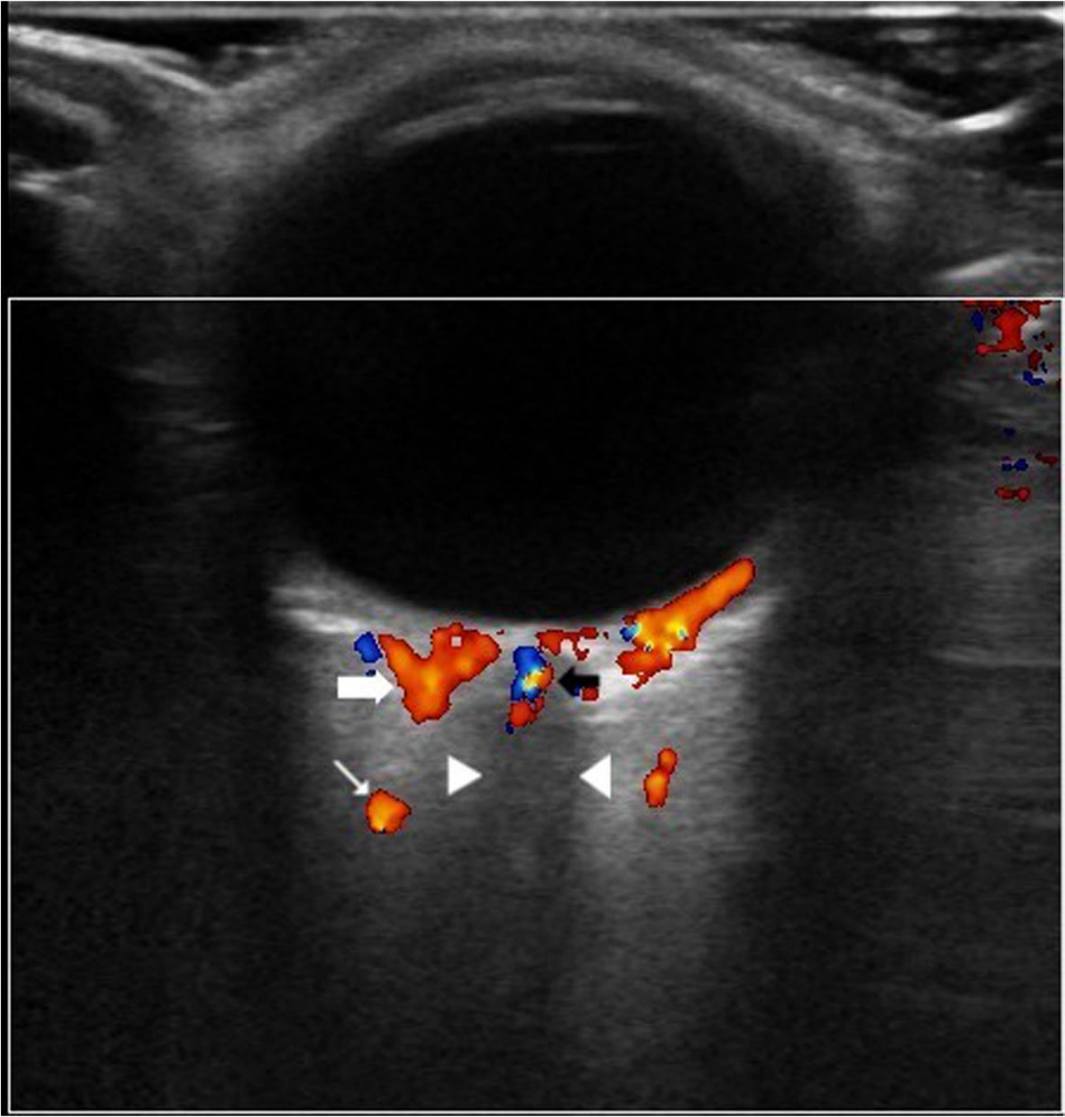
Axial doppler ultrasound section of left eye and orbit. Ophthalmic artery (white thin arrow), short posterior ciliary artery (white thick arrow), central retinal artery-vein (black arrow) and optic nerve shadow (arrowheads) can be seen posterior to the globe

All measurements were obtained by an experienced radiologist (HA) via Hitachi HiVision Preirus equipment (Tokyo, Japan) using a 7.5-MHz phased array linear probe with ultrasonic transmission gel on the upper eyelid. The evaluation of CDU of ophthalmic artery (OA), posterior ciliary artery (PCA) and central retinal artery (CRA) were performed at supine position and upright gaze behind the closed eyes as described by Lieb et al. [8]. Optic nerve shadow was used as a guide for the recognition of the posterior orbital vessels during the sonography (Fig. 1). No angle correction required due to alignment of the axis of the measured vasculature with the sound beam [9]. Peak systolic blood flow velocity (PSV) and end-diastolic blood flow velocity (EDV) values of OA, PCA and CRA were measured automatically in centimeters per second by the imaging device following the visualization of all arteries. Resistive index (RI) and pulsatility index (PI), which reflect vascular resistance, were automatically calculated for OA, PCA and CRA by the device.

Detailed ophthalmic examination, CDU and SD-OCT imaging were performed before surgery, and repeated at 1st week and 1st month after surgery. All CDU and SD-OCT evaluations were obtained between 09.00 and 12.00 a.m. to exclude diurnal variation. Preoperative examinations were masked according to whether the patient was in Group 1, 2, or control group. Postoperative evaluations could not be masked in Group 1 and 2 because of conjunctival hyperemia.

### Statistical analysis

Statistical analysis was performed via Statistical Package for Social Sciences version 25.0 programme (SPSS Inc., Chicago, IL, USA). Histogram graph and Kolmogorov–Smirnov test were used to evaluate whether the variables were normally distributed. According to the normality of distribution, data were given as frequency (percentage) for categorical variables and as mean ± standard deviation for continuous variables. Categorical variables were compared with Chi-Square test. Kruskal Wallis test was used to examine non-parametric variables between two groups. The correlation between each parameter was analyzed using Spearman’s correlation test. Friedman test was used to determine the differences within the groups, and repeated-measures analysis was used to identify the differences between groups. The level of statistical significance was set at a *P-*value of less than 0.05.

## Results

A total of 26 patients who underwent horizontal rectus muscle surgery were enrolled in this study. The mean age of all patients was 8.82±2.49 (range: 5–15) years. Group 1 included 14 eyes of 14 patients [5 male (36%), 9 female (64%)] with a mean age of 9.01±2.74 years. Group 2 consisted of 12 patients [7 male (58%), 5 female (42%)] with a mean age of 8.55±2.33 years. Control group included 15 healthy individuals [5 male (33%), 10 female (67%)] with a mean age of 8.74±2.52 years. The age and sex distribution were similar between the 3 groups.

Early postoperative examinations revealed a few clinical findings including mild eyelid edema, subconjunctival hemorrhage, conjunctival hyperemia and mild chemosis related to the surgical procedure. These findings resolved spontaneously without any sequela within the first week after surgery. No major complications such as scleral perforation, ASI, hypotonia, intraocular hemorrhage or fat prolapsus were observed during or after surgery.

The preoperative blood flow velocity parameters were statistically similar in Group 1, Group 2 and control group (*p* > 0.05). However, there were significant differences in some parameters at postoperative measurements. The mean OA RI increased significantly at 1st week and 1st month after surgery compared to the preoperative values in Group 1 and Group 2 (Table 1; *P* = 0.029 and *P* = 0.045, respectively).

**Table 1:** Blood flow velocities (cm/s) in the ophthalmic artery among the groups

Parameter	Group 1 (*n* = 14)	Group 2 (*n* = 12)	Control (*n* = 15)	P*
PSV				
Preoperative	50.92 ± 14.34	55.63 ± 15.42	56.78 ± 8.54	
Postop 1st week	50.16 ± 9.95	60.15 ± 19.96	56.29 ± 8.45	0.712
Postop 1st month	50.62 ± 10.41	57.71 ± 10.02	56.04 ± 7.03	
P^**^ (within groups)	0.662	0.486	0.975	
EDV				
Preoperative	11.29 ± 6.82	11.42 ± 6.64	9.22 ± 4.45	
Postop 1st week	8.48 ± 3.63	10.61 ± 5.85	8.41 ± 42.03	0.678
Postop 1st month	9.54 ± 6.04	10.44 ± 6.05	8.82 ± 3.92	
P^**^ (within groups)	0.067	0.717	0.407	
RI				
Preoperative	0.79 ± 0.07^a^	0.79 ± 0.11^c^	0.83 ± 0.09	
Postop 1st week	0.83 ± 0.07^b^	0.81 ± 0.12^d^	0.85 ± 0.08	0.760
Postop 1st month	0.82 ± 0.08^b^	0.82 ± 0.09^d^	0.84 ± 0.08	
P^**^ (within groups)	**0.029**	**0.045**	0.641	
PI				
Preoperative	1.86 ± 0.48	1.94 ± 0.81	2.15 ± 0.63	
Postop 1st week	2.01 ± 0.44	2.04 ± 0.61	2.21 ± 0.58	0.976
Postop 1st month	2.12 ± 0.54	2.11 ± 0.53	2.33 ± 0.81	
P^**^ (within groups)	0.390	0.423	0.717	

Data are given as mean ± standard deviation. Same letters denote the lack of statistically significant differences within groups measurements

*Postop* Postoperative, *PSV* Peak systolic velocity, *EDV* End-diastolic velocity, *RI* Resistive index, *PI* Pulsatility index

^*^Repeated measures analysis of variance (between groups)

^**^Friedman test

There was a significant increase in the mean PCA PSV at 1st postoperative week compared to the preoperative value in Group 1 (Table 2; *P* = 0.002). A slight but insignificant increase was also detected in the mean EDV, RI and PI of PCA at 1st week after surgery compared to preoperative values in Group 1.

**Table 2:** Blood flow velocities (cm/s) in the posterior ciliary artery among the groups

Parameter	Group 1 (*n* = 14)	Group 2 (*n* = 12)	Control (*n* = 15)	P^*^
PSV				
Preoperative	21.08 ± 3.49^a^	23.95 ± 4.38	23.41 ± 5.86	
Postop 1st week	24.64 ± 2.71^b^	22.92 ± 4.04	24.15 ± 5.62	0.119
Postop 1st month	20.81 ± 3.67^a^	21.42 ± 6.12	23.35 ± 5.52	
P^**^ (within groups)	**0.002**	0.183	0.926	
EDV				
Preoperative	6.44 ± 2.73	7.09 ± 2.33	8.09 ± 3.24	
Postop 1st week	6.86 ± 1.42	6.28 ± 2.52	7.69 ± 3.14	0.815
Postop 1st month	5.99 ± 1.75	6.29 ± 2.84	7.26 ± 3.34	
P^**^ (within groups)	0.230	0.320	0.323	
RI				
Preoperative	0.69 ± 0.11	0.71 ± 0.09	0.66 ± 0.12	
Postop 1st week	0.72 ± 0.05	0.72 ± 0.09	0.69 ± 0.09	0.962
Postop 1st month	0.72 ± 0.07	0.71 ± 0.08	0.69 ± 0.07	
P^**^ (within groups)	0.790	0.850	0.974	
PI				
Preoperative	1.15 ± 0.26	1.31 ± 0.35	1.14 ± 0.32	
Postop 1st week	1.27 ± 0.25	1.24 ± 0.26	1.18 ± 0.22	0.576
Postop 1st month	1.22 ± 0.21	1.22 ± 0.19	1.11 ± 0.19	
P^**^ (within groups)	0.230	0.529	0.497	

Data are given as mean ± standard deviation. Same letters denote the lack of statistically significant differences within groups measurements

*Postop* Postoperative, *PSV* Peak systolic velocity, *EDV* End-diastolic velocity, *RI* Resistive index, *PI* Pulsatility index

^*^Repeated measures analysis of variance (between groups)

^**^Friedman test

Apart from these increased parameters, there were no significant differences within and among the groups regarding the preoperative and postoperative blood flow velocity measurements of OA, PCA and CRA in Group 1 and Group 2 (Tables 1, 2, 3; *P* > 0.05). In the control group, blood flow velocity parameters of OA, PCA and CRA were similar before and after surgery (Tables 1, 2, 3; *P* > 0.05). Additionally, there were no significant differences in blood flow velocity parameters of OA, PCA and CRA between Group 1, Group 2 and control group at all time points (Tables 1, 2, 3; *P* > 0.05).

**Table 3:** Blood flow velocities (cm/s) in the central retinal artery among the groups

Parameter	Group 1 (*n* = 14)	Group 2 (*n* = 12)	Control (*n* = 15)	P^*^
PSV				
Preoperative	14.36 ± 2.55	13.55 ± 3.51	14.05 ± 2.23	
Postop 1st week	15.51 ± 3.59	14.92 ± 3.21	14.21 ± 2.6	0.921
Postop 1st month	14.96 ± 3.35	14.18 ± 2.98	14.09 ± 1.97	
P^**^ (within groups)	0.402	0.761	0.670	
EDV				
Preoperative	4.45 ± 1.62	4.22 ± 1.45	4.96 ± 1.47	
Postop 1st week	4.27 ± 1.03	4.33 ± 1.61	4.41 ± 1.15	0.869
Postop 1st month	4.39 ± 1.15	4.13 ± 1.16	4.41 ± 1.13	
P^**^ (within groups)	0.898	0.643	0.199	
RI				
Preoperative	0.71 ± 0.07	0.71 ± 0.04	0.65 ± 0.07	
Postop 1st week	0.72 ± 0.05	0.71 ± 0.06	0.68 ± 0.04	0.800
Postop 1st month	0.71 ± 0.05	0.72 ± 0.04	0.69 ± 0.06	
P^**^ (within groups)	0.641	0.975	0.264	
PI				
Preoperative	1.13 ± 0.21	1.15 ± 0.16	1.02 ± 0.17	
Postop 1st week	1.19 ± 0.18	1.16 ± 0.18	1.12 ± 0.12	0.830
Postop 1st month	1.16 ± 0.15	1.19 ± 0.14	1.05 ± 0.15	
P^**^ (within groups)	0.252	0.739	0.273	

Data are given as mean±standard deviation

*Postop* Postoperative, *PSV* Peak systolic velocity, *EDV* End-diastolic velocity, *RI* Resistive index, *PI* Pulsatility index

^*^Repeated measures analysis of variance (between groups)

^**^Friedman test

In Group 1 and Group 2, no significant change was found in the mean subfoveal CT at 1st week and 1st month after surgery compared to preoperative measurements (Table 4; *P* = 0.713 and *P* = 0.164, respectively). The analysis between the groups showed no statistically significant differences in the mean subfoveal CT among the control and patient group at all time points (Table 4; *P* > 0.05).

**Table 4:** Preoperative and postoperative subfoveal choroidal thickness among the groups

Choroidal thickness (μm)	Group 1 (*n* = 14)	Group 2 (*n* = 12)	Control (*n* = 15)	P^*^
Preoperative	335.94 ± 52.24	326.64 ± 54.78	338.42 ± 70.34	0.926
Postop 1st week	338.41 ± 47.61	325.55 ± 47.16	337.71 ± 68.32	0.874
Postop 1st month	335.18 ± 51.07	312.64 ± 51.05	338.72 ± 68.79	0.695
P^**^ (within groups)	0.713	0.164	1.000	

Data are given as mean ± standard deviation

^*^Kruskal–Wallis test (between groups)

^**^Friedman test

The examination of relationship between the percentage changes of blood flow velocity parameters and CT at 1st month after surgery in patients who underwent strabismus surgery revealed a negative correlation between the percentage changes of CT and CRA EDV only in Group 2 (Table 5; *P* = 0.011). There was no statistically significant correlation between CT and other blood flow velocity parameters of OA, PCA and CRA (Table 5; *P* > 0.05).

**Table 5:** Correlation analysis between blood flow velocities and CT parameters in groups (the percentage of change)

		CT
Parameter	Group 1	Group 2
OA PSV	*r*-0.196	0.045
	p* 0.450	0.894
OA EDV	*r*-0.249	0.355
	p* 0.335	0.285
OA RI	*r *0.441	-0.251
	p* 0.076	0.457
OA PI	*r *0.466	-0.309
	p* 0.059	0.355
PCA PSV	*r*-0.064	-0.264
	p* 0.808	0.433
PCA EDV	*r*-0.417	0.509
	p* 0.096	0.110
PCA RI	*r *0.423	-0.482
	p* 0.090	0.133
PCA PI	*r *0.384	-0.355
	p* 0.128	0.285
CRA PSV	*r*-0.139	-0.600
	p* 0.596	0.051
CRA EDV	*r*-0.074	-0.727
	p* 0.779	**0.011**
CRA RI	*r *0.087	-0.059
	p* 0.739	0.863
CRA PI	*r*-0.017	-0.209
	p*0.948	0.537

*CT* Choroidal thickness, *PSV* Peak systolic velocity, *EDV* End-diastolic velocity, *RI* Resistive index, *PI* Pulsatility index, *OA* Ophthalmic Artery, *PCA* Posterior Ciliary Artery, *CRA* Central Retinal Artery

^*^Spearman correlation analysis

## Discussion

Anterior ciliary arteries that provide at least 70–80% of the blood supply to the anterior segment are disrupted during a conventional strabismus surgery [3]. Interruption of the anterior segment circulation may result in ASI, a serious complication that may occur following strabismus surgery [1]. A previous experimental study showed that two or three rectus muscle recession in different combinations simultaneously may cause transient mild to moderate ASI, whereas simultaneous recession of four rectus muscles can result in severe ASI [10]. Children have a lower risk of ASI development, regardless of the number of disinserted rectus muscles during strabismus surgery [11]. Previous reports indicated that children developed ASI only in unusual clinical settings such as malnutrition, congenital ocular malformation, previous orbital surgery, and cicatricial retinopathy of prematurity [11]. However, Tibrewal et al. reported a case of 9-year-old healthy male who developed ASI after three rectus muscle surgery for congenital 6th nerve palsy [12].

Previous studies investigating retrobulbar hemodynamics after strabismus surgery included patients with a wide range of age [5, 6, 13]. In this study, we included only individuals aged 5 to 15 years to evaluate the alterations in retrobulber hemodynamics in patients not at risk of ASI. Because Tibrewal et al. reported a pediatric patient with ASI after three rectus muscle surgery, we excluded children who had more than two rectus muscle surgery [12]. The patients who had oblique or vertical rectus muscle surgery were also excluded because the risk of ASI has been shown to increase after strabismus surgery involving vertical muscles [2]. Additionally, none of our patients had a history of systemic disease or previous ocular trauma. We aimed to create a study group with as lower ASI risk as possible to accurately evaluate the changes in retrobulbar circulation.

In the literature data, there are previous studies evaluating postoperative alterations in retrobulbar blood flow using CDU in patients who underwent strabismus surgery [5, 6, 13]. In a previous study investigating the hemodynamic changes only in OA via CDU following double horizontal rectus muscle surgery, Bayramlar et al. found no difference between preoperative and postoperative OA blood flow parameters at 1st week and 1st month after surgery [14]. In another study, Güven et al. evaluated the changes in OA, CRA and PCA circulation before and 2–7 days after one or two horizontal rectus muscle surgery using CDI and found significantly higher values of indices and blood flow velocity parameters in all arteries after strabismus surgery [5]. The differences were more evident in patients who underwent double horizontal rectus muscle surgery. Güven et al. suggested that the reason for this evident difference was the doubled surgical irritation leading to more neurological stimulation in patients who had two horizontal rectus muscle surgery [5]. Additionally, the patients older than 15 years of age had strabismus surgery under retrobulbar anesthesia and akinesia, in Güven et al.’s study. The retrobulbar anesthesia itself, preferred anesthetic agent, increased surgical irritation, or surgery anxiety may have contributed to early postoperative alterations in retrobulbar hemodynamics, in their study. Akyüz Ünsal et al. evaluated blood flow changes in OA, CRA, and PCA using CDU on the 1st day and 1st month after strabismus surgery under general anesthesia, similar to our study design [6]. However their study groups consisted of patients with various types of strabismus including esotropia, exotropia, trochlear nerve palsy and oculomotor nerve palsy. They also performed adjustable suture surgery in half of the patients. Despite these various variables, Akyüz Ünsal et al. found no significant differences between preoperative and postoperative measurements of blood flow velocity parameters and indices among the groups [6]. In a previous study, Pelit et al. investigated the alterations in retrobulbar hemodynamics via CDU on the 1st and 7th postoperative day after single and double horizontal muscle surgery and found an increase in PSV and EDV of OA whereas a decrease in RI and PI of OA on the 1st postoperative day [13]. These differences were more prominent in patients who underwent double horizontal rectus muscle surgery. Retrobulbar blood flow velocity parameters and indices returned to baseline values at the end of 1st week in that study. Pelit et al. reported that increased demand of anterior segment resulted in early postoperative alterations in retrobulbar circulation and these changes indicated the increased blood flow and decreased vascular resistance which could be expected in inflammatory conditions [13]. They also commented that the return to the normal velocity and resistance parameters at 1st week after surgery might be a sign of decreased inflammation [13]. Contrary to Pelit et al.’s study indicating a decreased OA RI in the early postoperative period, the mean OA RI increased significantly at 1st week and 1st month after single and double horizontal rectus muscle surgery in our study. We also found an increase in the blood flow velocity parameters of PCA at 1st week after single horizontal rectus muscle surgery but there were no other alterations within and among the groups regarding the preoperative and postoperative OA, PCA and CRA measurements. The release of local vasoconstrictive factors leading to vasoconstriction may be the cause of increase in OA RI, PCA RI and PCA PI. The increased PCA PSV and PCA EDV may also be a result of increased blood flow, in our study. Moreover there was no significant correlation between CT and other blood flow velocity parameters of OA, PCA and CRA, except for the negative correlation between the percentage changes of CT and CRA EDV only in patients who underwent double horizontal rectus muscle surgery. The reason for the lack of correlation between CDU parameters and CT may be that there was no difference between the mean preoperative and postoperative subfoveal CT among the 3 groups, in our study.

Our study is quite different from previous studies in the literature. Unlike Bayramlar et al.’s study evaluating blood flow only in OA after strabismus surgery, we investigated all potentially affected arteries in our study. Moreover we operated all patients under general anesthesia compared to the study of Güven et al. in which they performed retrobulbar anesthesia for strabismus surgery. Akyüz et al.’s study including patients with different strabismus types, vertical rectus muscle surgery, adjustable suture technique and limbal conjunctival incisions, is also quite different from our study. Finally, none of these studies investigated the correlation between retrobulbar blood flow changes and CT. There are also studies evaluating CT but not retrobulbar blood flow after strabismus surgery [15]. Inan et al. showed a significant decrease in subfoveal CT on the 1st day and 2nd week after single horizontal rectus muscle surgery, but they did not find a significant change in subfoveal CT after double horizontal rectus muscle surgery. They hypothesized that single horizontal rectus muscle surgery may cause vascular damage, compensatory vasoconstriction and ischemia in the choroid rather than inflammation whereas multiple rectus muscle surgery may cause more choroidal inflammation resulting in increased choroidal blood flow [15]. Although Inan et al. commented on choroidal blood flow, they did not correlate their findings with retrobulbar blood flow using CDU. The alterations in retrobulbar hemodynamics may also affect choroidal circulation, leading to changes in CT. Our study is the first in the literature to evaluate the changes in retrobulbar circulation as well as the relationship between these changes and CT.

Previous reports suggested that fornix-based conjunctival incisions cause less ASI compared to limbus-based incisions because fornix-based incisions preserve the perilimbal conjunctiva-Tenon’s capsule junction and episcleral vessels [1]. It was also suggested that using a limbal conjunctival incision during strabismus surgery may result in greater postoperative inflammation [16]. Pelit et al. did not specify which technique they used for the conjunctival incision [13]. Akyüz Ünsal et al. used limbal conjunctival incisions and recessed the conjunctiva approximately 4 mm from the limbus in the adjustable suture surgery [6]. In our study, all conjunctival incisions were made with a fornix-based approach to avoid the inflammatory effect of limbal incision and to minimize the risk of ASI.

Although widely used in ophthalmology to investigate orbital and ocular blood flow characteristics, CDI has also some disadvantages. Akyüz Ünsal et al. reported that CDU is a time-consuming examination and has a user-dependent interpretation [6]. Pelit et al. stated that performing CDI in the early postoperative period was difficult due to patient discomfort, especially in children [13]. However CDI is a noninvasive and beneficial imaging method that provides a reproducible qualitative and quantitative information about retrobulbar blood flow in a variety of ophthalmologic disorders [13].

The current study has also some limitations. All measurements and evaluations including CDU and choroidal imaging were performed at 1st week and 1st month after strabismus surgery, but could not be performed on the 1st day after surgery due to patients’ discomfort. The lack of measurements on the 1st postoperative day may have been insufficient to reflect early postoperative alterations in retrobulbar circulation and CT. Additionally, we could not correlate our findings with optical coherence tomography angiography (OCTA) since we do not have an OCTA in our clinic. The small number of cases was also a limitation of this study. Further studies with larger numbers of objects using OCTA are needed to understand the hemodynamic changes following strabismus surgeries.

## Conclusions

Single and double horizontal rectus muscle surgeries cause some alterations in retrobulbar hemodynamics but these changes do not correlate with CT. CDU can be used to determine the risk of ASI and take preventive measures in patients scheduled for strabismus surgery.

## Data Availability

The datasets generated and/or analyzed during the current study are not publicly available due to local data protection laws but are available from the corresponding author on reasonable request.
